# Novel 4D-MRI of tumor infiltrating vasculature: characterizing tumor and vessel volume motion for selective boost volume definition in pancreatic radiotherapy

**DOI:** 10.1186/s13014-018-1139-2

**Published:** 2018-10-01

**Authors:** Wensha Yang, Zhaoyang Fan, Zixin Deng, Jianing Pang, Xiaoming Bi, Benedick A Fraass, Howard Sandler, Debiao Li, Richard Tuli

**Affiliations:** 1 Department of Radiation Oncology, Cedars Sinai Medical Center, 8700 Beverly Blvd., Los Angeles, CA 90048 USA; 2 Department of Biomedical Sciences, Biomedical Imaging Research Institute, Cedars Sinai Medical Center, Los Angeles, CA USA; 3 Department of Bioengineering, University of California, Los Angeles, Los Angeles, CA USA; 4 Siemens Healthineers, Los Angeles, USA

## Abstract

**Background:**

Pancreatic ductal adenocarcinoma has dismal prognosis. Most patients receive radiation therapy (RT), which is complicated by respiration induced organ motion in upper abdomen. The purpose of this study is to report our early clinical experience in a novel self-gated k-space sorted four-dimensional magnetic resonance imaging (4D-MRI) with slab-selective (SS) excitation to highlight tumor infiltrating blood vessels for pancreatic RT.

**Methods:**

Ten consecutive patients with borderline resectable or locally advanced pancreatic cancer were recruited to the study. Non-contrast 4D-MRI with and without slab-selective excitation and 4D-CT with delay contrast were performed on all patients. Vessel-tissue CNR were calculated for aorta and critical vessels (superior mesenteric artery or superior mesenteric vein) encompassed by tumor. Respiratory motion trajectories for tumor, as well as involved vessels were analyzed on SS-4D-MRI. Intra-class cross correlation (ICC) between tumor volume and involved vessels were calculated.

**Results:**

Among all 4D imaging modalities evaluated, SS-4D-MRI sampling trajectory results in images with highest vessel-tissue CNR comparing to non-slab-selective 4D-MRI and 4D-CT for all patients studied. Average (±standard deviation) CNR for involved vessels are 13.1 ± 8.4 and 3.2 ± 2.7 for SS-4D-MRI and 4D-CT, respectively. The ICC factors comparing tumor and involved vessels motion trajectories are 0.93 ± 0.10, 0.65 ± 0.31 and 0.77 ± 0.23 for superior-inferior, anterior-posterior and medial-lateral directions respectively.

**Conclusions:**

A novel 4D-MRI sequence based on 3D-radial sampling and slab-selective excitation has been assessed for pancreatic cancer patients. The non-contrast 4D-MRI images showed significantly better contrast to noise ratio for the vessels that limit tumor resectability compared to 4D-CT with delayed contrast. The sequence has great potential in accurately defining both the tumor and boost volume margins for pancreas RT with simultaneous integrated boost.

## Introduction

Pancreatic ductal adenocarcinoma (PDA) has the worst outcome of any solid tumor [1]. Whereas surgical resection remains the mainstay therapy, 80% of patients with non-metastatic disease have unresectable tumors that are unlikely to be down-staged after standard chemo-radiation therapy, due to the geometric relationship of the primary tumor to the surrounding vasculature [2]. Nevertheless, the overall survival rates in the patient group undergoing margin negative resection after neoadjuvant therapy are 2–3 times of the unresectable patient group [3, 4]. Stereotactic body radiation therapy (SBRT) has shown to improve local tumor control rates, yet has disappointing down-staging rates. Alternatively, radiation therapy (RT) with simultaneous integrated boost (SIB) to cancerous tissue surrounding tumor infiltrating vasculature has the potential to sterilize tumor around the vessels that have precluded resectability [5, 6]. However there are significant technical challenges associated with accurately escalating (boosting) radiation dose to the vessel/tumor interface due to substantial internal organ motion in the upper abdominal region. [7–10] SBRT with SIB to tumor/vessel interface is further complicated by the fact that the motion of the cancerous tissue surrounding the tumor infiltrating vessels may be different from center of the pancreas tumor [11]. Per standard clinical practice in our institution, a free-breathing (FB) contrast enhanced helical CT and a four dimensional CT (4D-CT) are performed to quantify the motion. Depending on patient compliance, 4D-CT can either be performed right after FB-CT with a sufficient amount of contrast left over in the blood vessels to enhance vasculature visibility, or after a long breathing coaching session to reach a stable respiration pattern required for a successful 4D-CT acquisition, when the contrast in the region of interest has already diminished. Risks associated with radiation dose and contrast agents also prevent this procedure from being repetitively performed on patients. [12, 13] The problem is further compounded by poor CT soft tissue contrast and 4D-CT stitching artifacts [14]. For these reasons, 4D-CT with delayed intravenous and oral contrasts often has limited value to evaluate soft tissue and blood vessel respiratory motion.

Recently the interest in using magnetic resonance imaging (MRI) in pancreas radiotherapy has increased. The superior soft tissue contrast and versatile imaging sequences of MRI can facilitate margin definitions in SBRT-SIB. Frequent imaging procedures necessary for organ motion characterization are also impeded by radiation dose from 4D-CT while not an issue in MRI. Since current MRI speed is insufficient to capture the three-dimensional motion of upper abdominal organs in real time, four dimensional MRI (4D-MRI) was developed to reconstruct motion encoded images from multiple breathing cycles. Early 4D-MRI was reconstructed by sorting 2D cine images from consecutive slices, thus its quality was degraded by severe stitching artifacts [15–17]. The artifacts were eliminated with new 4D-MRI techniques based on 3D acquisition sequences [18] and k-space sorting such as the recently developed self-gated 4D-MRI technique with 3D radial sampling and k-space sorting [16, 19]. The new class of 4D-MRI sequences also provides high isotropic resolution that was unachievable with 2D cine based 4D-MRI.

Recently, slab-selective excitation was proposed to improve vessel-tissue contrast and overall image quality in 3D radial-sampling-based 4D-MRI [20]. This approach exploits the in-flow effect. As fresh blood first enters the imaging volume of interest and experiences fewer RF-pulses than stationary tissues, blood signal is markedly higher than that of tissue, creating appreciable vessel-tissue contrast for various types of cancers including the pancreatic cancer. However, its ability of quantifying the pancreatic tumor and tumor infiltrating vessel motion has not been studied and compared with the current state of the art method 4D-CT.

To quantify this ability, the current study aims to exploit the technological potential of contrast free vessel highlighting in combination with the high resolution 4D-MRI method for a pancreatic patient cohort. The goal is to show the reproducibility and consistency of this novel 4D-MR method. Through the comparison with 4D-CT, we specifically evaluate the respiratory motion trajectories for both the tumor and the tumor infiltrating blood vessels that used to define potential boost volumes in the pancreas SIB treatment.

## Methods and materials

### Patients

Ten consecutive patients (seven males and three females, average age of 65) diagnosed with locally advanced, borderline resectable or locally recurrent PDA were recruited for the study under a protocol approved by the institutional review board. Gross tumor volumes range from 14 to 220 cc (mean ± σ = 86 ± 56 cc).

### Imaging studies

The 4D-MRI sequence was described in details in previous publications [16]. In short, an in-house developed, RF and spoiled gradient recalled echo (GRE) sequence with Koosh-Ball (KB) 3D k-space radial-sampling trajectory, 1D self-gating and slab-selective (SS) excitation was implemented at 3 T (Biograph mMR, Siemens Healthineers. USA). Amplitude based sorting was applied in k-space before image reconstruction. This sequence is denoted as SS-4D-MRI. KB 4D-MRI with non-selective excitation was also performed and denoted as NS-4D-MRI. The shared imaging protocol for SS and NS were as follows: field of view (FOV) = (400 mm) [3]; prescribed spatial resolution =1.56 mm; flip angle = 12^o^; repetition time (TR)/echo time (TE) = 5.5/2.68 ms; readout bandwidth = 429 Hz/pixel; fat suppression with water excitation on; total scan time = 5 min. Patients were lying on the imaging couch in the head first supine position, with arms placed on the side for the comfort. No special immobilization was used.

During the patient’s clinical CT simulation visit, a standard contrast enhanced FB-CT scan was first performed on a 16-slice scanner (Optima CT580; GE Healthcare, Milwaukee, WI), followed by a 4D-CT scan. Cine mode was used for the 4D-CT with the following parameters: 120 kV, variable mA, gantry rotation period of 1 s, and slice thickness of 2.5 mm. The cine duration was set to be the patient’s breathing period plus 1.5 s. A total of 3000 images were set as the limit, which typically covers the region from above the diaphragm down to below the distal portion of the kidneys. 4D-CT images were then retrospectively binned using phase based sorting method in AdvantageSim™ 4D software (GE Healthcare, Milwaukee, WI). Variable time gaps ranging from 5 min to 30 min between the first contrast FB-CT scan and the following 4D-CT scan had to be used depending on the individual patient’s compliance with the audio coaching of the respiration. Patients were immobilized in a vacuum lock bag and simulated in a head-first supine position with both arms raised above the head.

### Image analysis

Pancreatic tumor and the involving blood vessels were delineated on the end-of-exhalation image bin by a single radiation oncologist. A B-spline based image registration was performed on both the 4D-MRI and 4D-CT using VelocityAI™ (Varian Medical System, Palo Alto, CA). Contours were mapped to the other bins. Motion trajectories for both the tumor and the tumor infiltrating blood vessels were extracted by the coordinates of the geometric centers from each respiration bin. The correlation coefficient (CC) was calculated from the tumor and the involved vessel motion trajectories for each patient.

Vessel-tissue contrast to noise ratio (CNR) is defined as $CNR=\frac{\left|{S}_V-{S}_P\right|}{\sigma_L}$ in which *S*_*V*_ is the average intensity of a selected region in the vessel, S_P_ is the average intensity of the adjacent region in the pancreas tumor and σ_L_ is the standard deviation of a relatively homogeneous region in the liver. Two CNRs, the aorta (CNR_aorta_) and tumor infiltration blood vessel (CNR_IV_), were calculated on both the 4D-MRI and 4D-CT.

## Results

SS-4D-MRI was successfully implemented on 10 pancreatic cancer patients. All scans resulted consistent and satisfactory imaging quality.

**Fig. 1:**
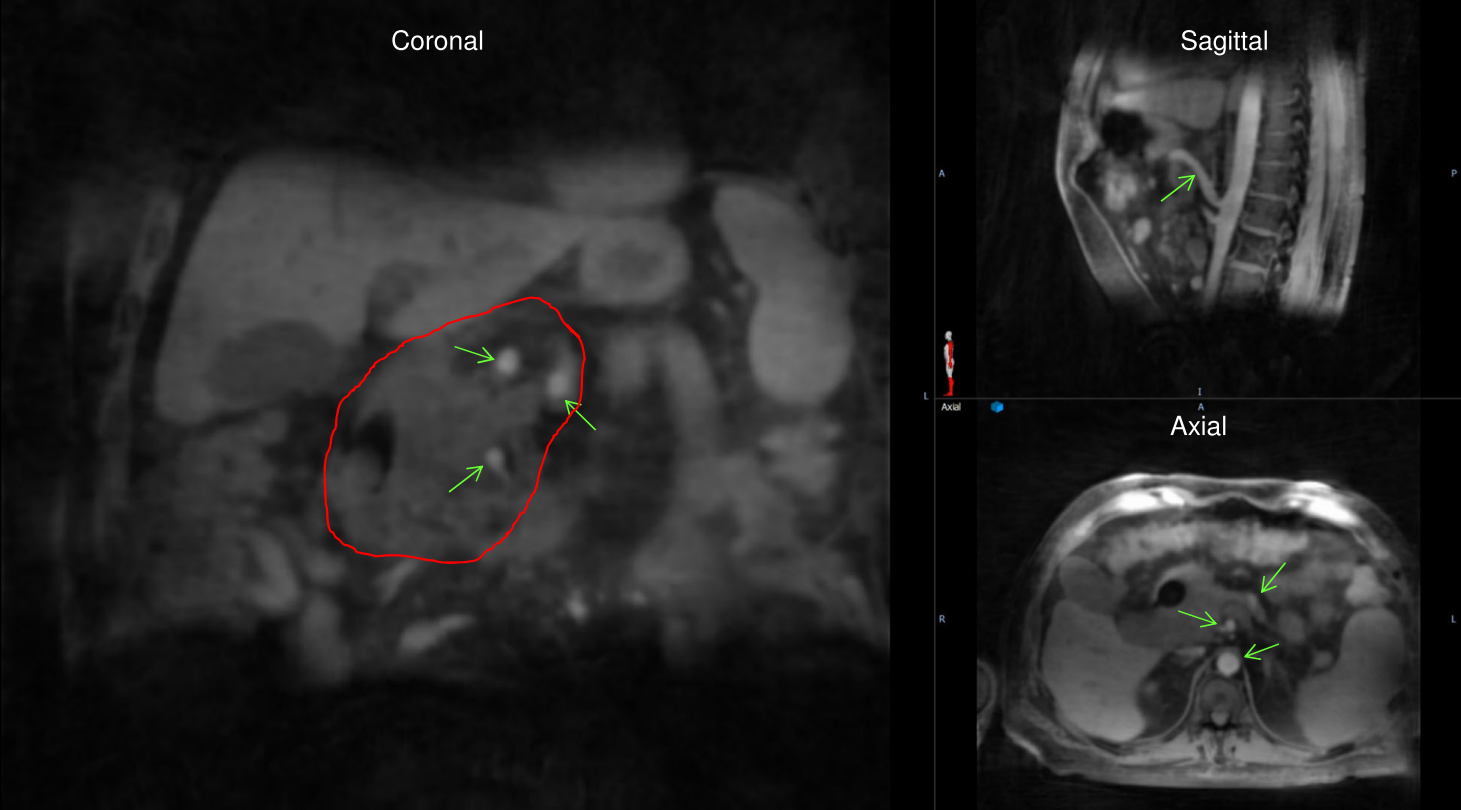
Example 4D-MRI images in coronal, sagittal and axial planes showing the tumor (red circle) and infiltrated blood vessels (green arrows)

Figure 1 shows an example patient’s SS-4D-MRI in three cardinal planes (coronal, sagittal and axial). Excellent image quality and high isotropic image resolution (1.6 mm) are achieved in all planes. Fine image features such as blood vessels in the liver, stomach wall, diaphragm and bifurcation of the blood vessels from the great vessels are all visible in 4D-MRI images. Red circles show the tumor region and green arrows show the tumor infiltrating blood vessels. Excellent vessel contrast was also observed in all three planes.

**Fig. 2:**
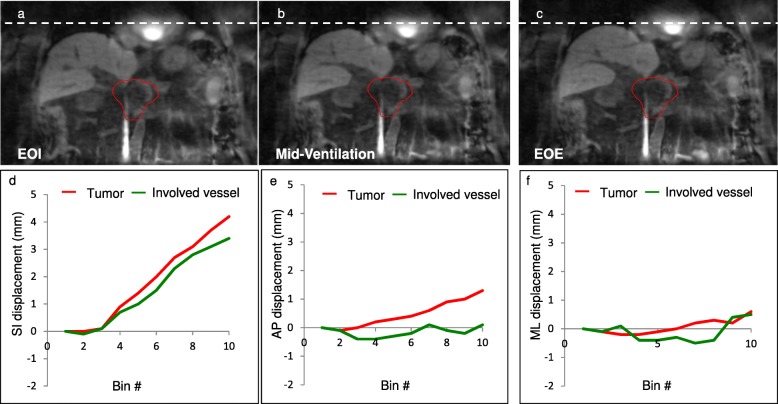
Example 4D-MRI coronal images at (**a**). end-of-inhalation (EOI), (**b**). mid-ventilation, and (**c**). end-of-exhalation (EOE) bins; (**d-f**) show the motion trajectories derived from the tumor (red) and involved vessel (green) in the superior-inferior, anterior-posterior and medial-lateral directions

Figure 2 shows an example patient’s SS-4D-MRI in the coronal plane at end-of-inhalation (EOI), mid-ventilation and end-of-exhalation (EOE) bins (Fig. 2a-c), respectively. The patient’s diaphragm motion is readily visible using a white dashed straight line as the reference. Motion trajectories extracted from the center of mass coordinates for both the tumor and involved vessel contours were plotted for the superior-interior (SI), anterior-posterior (AP) and medial-lateral (ML) directions (Fig. 2d-f). For this patient, tumor and involved vessel movements correlate well in the SI direction, but less well in the AP and ML directions, possibly due to the small motion amplitudes (< 1.6 mm, the image resolution) observed in these two directions.

**Fig. 3:**
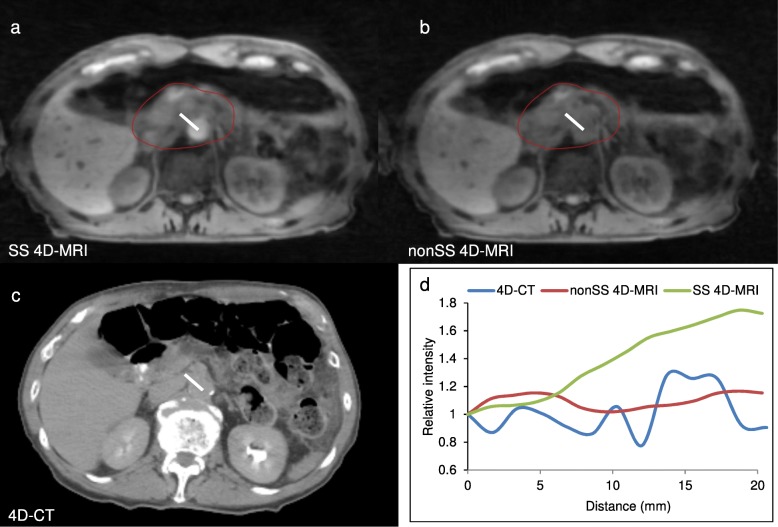
Example images from slab-selective 4D-MRI (**a**), non-slab-selective 4D-MRI (**b**) and 4D-CT (**c**) for the same patient. Red circle indicates the tumor region and green arrow indicates the involved blood vessel. **d** The relative image intensity profile across the tumor and aorta interface indicated by the white line on the 4D-CT image in (**c**), with slab-selective-4D-MRI showing higher contrast across the tumor/vessel interface than that non-slab-selective-4D-MRI and 4D-CT

Figure 3 shows the comparisons of SS-4D-MRI (a) to NS-4D-MRI (b) and 4D-CT (c) for an example patient. 4D-MRI with slab selective excitation clearly enhances the imaging signal and improved the vessel CNR, compared to NS-4D-MRI. SS-4D-MRI also results in visually improved vessel CNR compared to 4D-CT. Relative image intensity profiles (as shown in Fig. 3d, normalized to the starting of the profile for each imaging technique), also present the CNR enhancement in a quantitative manner. The white straight line on the CT (3c) shows the image region for the plotted profiles.

Table 1 summarizes basic clinical information, the vessel-tissue CNR and correlation coefficients calculated from motion trajectories of involved vessel and from tumor for the patient cohort. Quantitative analysis showed significantly improved *CNR*_*aorta*_ of 23.0 ± 18.3 (mean ± SD) on SS-4D-MRI from 2.1 ± 2.0 on 4D-CT with delayed contrast with a *p* value of 0.002. *CNR*_*IV*_ also significantly improved from 3.2 ± 2.7 on 4D-CT to 13.1 ± 8.4 on SS-4D-MRI with a p value of 0.001. The mean correlation coefficients (mean ± SD) calculated from tumor and vessel motion trajectories were 0.93 ± 0.10, 0.65 ± 0.31 and 0.77 ± 0.23 in the SI, AP and ML directions respectively. Correlation coefficients for the SI direction are greater than 0.9 for all patients except two (CC = 0.81 and 0.69), which might be a result of local anatomical deformation of the tumor in the vessel regions or small motion amplitudes for these two patients. For this patient cohort, tumor moves more than involved vessels, with SI motion range of 3.6 ± 1.5 mm (mean ± SD) vs. 2.9 ± 2.1 mm for tumor and vessels respectively.

**Table 1:** Patient characteristics, motion range, comparison of contrast to noise ratio, and correlation coefficient of tumor and vessel motion trajectories

Patient ID	Gender	Age	GTV (cc)	GTV SI motion (mm)	IV SI motion (mm)	SS-4D-MRI / 4D-CT		ICC (PTV vs. involved vessels) on SS-4D-MRI		
						CNR aorta	CNR IV	SI	AP	ML
1	M	54	220	3.7	3.2	13.2 / 0.7	11.2 / 2.9	0.99	0.18	0.97
2	M	79	53	4.2	3.5	13.5 / 0.3	4.6 / 0.7	1.00	0.44	0.42
3	M	69	100	3.3	2.5	43.8 / 7.3	27.5 / 5.8	0.96	0.88	0.96
4	M	79	38	3.8	2.4	14.5 / 1.0	4.7 / 1.6	0.99	0.93	0.87
5	F	48	58	1.5	1.1	32.0 / 0.8	23.1 / 0.8	0.81	0.44	0.94
6	M	69	48	2.3	1.5	6.2 / 2.0	6.9 / 0.3	0.93	0.72	0.44
7	M	67	87	4.8	4.6	14.8 / 1.4	9.1 / 1.4	0.99	0.98	0.63
8	F	79	125	2.3	0.1	18.7 / 1.7	11.9 / 6.5	0.69	0.11	1.00
9	M	34	112	7.3	7.6	64.2/ 1.9	23.5 / 7.6	1.00	0.92	0.93
10	F	72	14	3.0	2.3	9.5 / 3.8	8.7 / 4.4	0.97	0.85	0.52
Average	n/a	65	86	3.6	2.9	23.0 / 2.1	13.1 / 3.2	0.93	0.65	0.77
Stdev	n/a	14.3	55.8	1.5	2.1	18.3 / 2.0	8.4 / 2.7	0.10	0.31	0.23
p	n/a	n/a	n/a	n/a	n/a	0.002*	0.001*	n/a	n/a	n/a

## Discussion

**Fig. 4:**
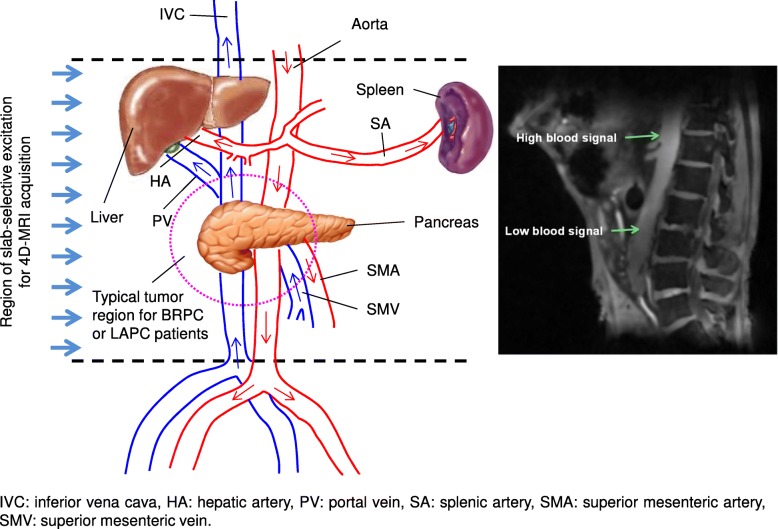
Schematic presentation of slab-selective excitation region and the local anatomies around pancreas with major vessels that limit the resectability. Magenta circle indicates the tumor region that normally seen for locally advanced or borderline resectable pancreatic cancer patients

Despite the poor prognosis, evidence suggests that pancreas tumor respond to sufficiently high radiation dose [21]. The dose is often excluded by sensitive nearby organs. As shown in Fig. 4, pancreas is surrounded by major blood vessels that supply nutrients and oxygen to the organs nearby. Tumors grown on the pancreas head and/or body usually wrap around these vessels such as superior mesenteric artery and superior mesenteric vein that prevent the surgical procedures. SBRT with SIB to the cancerous regions around the vessels can be designed to specifically sterilize the tumor non-invasively, although the complete control of the whole tumor is limited by the radiation dose tolerable by the normal critical organs nearby. Down-staging the patients after controlling the tumor around the vessels is the key to the success of the margin negative resection post radiation therapy. Therefore, radiation therapy with simultaneously integrated boost to the infiltrated blood vessels has attracted increasing interest for its potential to improve the resectability conversion rate and long-term disease free survival for the pancreas cancer patients who did receive subsequent margin negative resection. However, SIB of a moving target is challenging even with image guided radiation therapy (IGRT). Lack of image quality to differentiate soft tissue tumor and involved blood vessels in current clinical IGRT practice, which uses 4D-CT to evaluate pancreas tumor motion, calls for novel and advanced imaging strategies with high isotropic resolution, reduced imaging artifacts and improved soft tissue contrast. To our knowledge, this study is the first one that implements such a 4D-MRI sequence with the intent of characterizing not only the tumor motion, but also the motion of tumor infiltrating blood vessels. The key feature of this novel non-contrast 4D-MRI sequence is the fact that it can distinguish between motion of the vessels and the rest of the tumor. Another key feature is the ability to significantly improve the vessel conspicuity, which was extremely poor in non-vessel highlighting 4D-MRI or non-contrast 4D-CT images. The remarkable improvement in vessel contrast can help clinicians better identify and analyze the boost volume margin in the pancreas SBRT-SIB. The sequence uses slab-selective excitation during image acquisition with a 3D radial Koosh-Ball like sampling trajectory, which takes advantage of the fresh in-flow blood to enhance the blood signal. The vessel contrast enhancement only relies on the natural blood flow of the patients, so no additional contrast agent is needed. This feature makes frequent imaging possible for treatment strategies such as adaptive radiation therapy in which fast daily motion assessments prior to radiation dose delivery are preferred. Recent development in tumor response assessment using 4D imaging modalities potentially can also benefit from this novel 4D-MRI sequence with more frequent longitudinal 4D imaging data becoming available.

This study also contributes to the recent emergence of MRI-only simulation for radiotherapy treatment planning. Besides its superior soft-tissue contrast as compared to CT, other benefits of MRI including functional and dynamic imaging for tumor delineation and motion assessment without radiation dose are well acknowledged by the radiation therapy society. Both the tumor and involved Advancement in 4D-MRI sequence including our vessel highlighting 4D-MRI will facilitate the MR-only simulation being implemented in radiation therapy.

One drawback of this study is the imaging reconstruction speed. It takes around 8 h for our in-house developed program to reconstruct and post-process a KB 4D-MRI data set on a server equipped with 12-core Intel (Beaverton, OR) Xeon central processing unit and 96 GB of memory. The speed of image reconstruction and imaging processing needs to be significantly improved for wide clinical adaptation. There are several methods that can be implemented to speed up the image reconstruction. One example is to use fast iterative shrinkage-thresholding algorithm (FISTA), which preserves the computational simplicity of iterative shrinkage-thresholding algorithms but with a global rate of convergence which is proven to be significantly better theoretically and practically [22]. Working closely with the MRI vendors to implement the sequence will also help adapting the sequence with more practical reconstruction time in a real clinical setting.

## Conclusion

A novel 4D-MRI sequence based on 3D-radial sampling and slab-selective excitation has been assessed on pancreas cancer patients. The non-contrast 4D-MRI images showed significantly better contrast to noise ratio for the vessels that limit tumor resectability compared to 4D-CT with delayed contrast. The sequence has great potential in accurately defining both the tumor and boost volume margins for pancreas SBRT-SIB.

## Abbreviations

4D-CT: Four dimensional computed tomography
AP: Anterior-posterior
CNR: Contrast to noise ratio
EOE: End-of-exhalation
EOI: End-of-inhalation
FB-CT: Free breathing computed tomography
GRE: Gradient recalled echo
ML: Medial-lateral
MRI: Magnetic resonance imaging
NS: Non-selective
PDA: Pancreatic ductal adenocarcinoma
RT: Radiation therapy
SBRT: Stereotactic body radiation therapy
SI: Superior-inferior
SIB: Simultaneous integrated boost
SS: Slab-selective
